# Integrating psychological care in the treatment of chronic neuropathic pain and hemifacial spasm: a call for interdisciplinary approaches

**DOI:** 10.1007/s10143-024-02460-7

**Published:** 2024-05-21

**Authors:** Muhammad Hassan Khalid, Momina Khawaja, Haseeb Mehmood Qadri, Asif Bashir

**Affiliations:** 1 Quaid-E-Azam Medical College, Bahawalpur, Pakistan; 2 Gulf Medical University, Ajman, United Arab Emirates; 3 Department of Neurosurgery, Punjab Institute of Neurosciences, Lahore, Pakistan; 4 Department of Neurosurgery, Punjab Institute of Neurosciences, Lahore, Pakistan

Dear Editor,

Chronic neuropathic pain (CNP) is defined as pain persisting for over three months due to a disease or injury affecting the somatosensory nervous system [1]. This category includes disorders such as trigeminal neuralgia, hemifacial spasms, phantom limb syndrome, shingles, and diabetic neuropathy. Common risk factors associated with CNP encompass metabolic diseases, neurodegenerative diseases, tumors, infections, and psychosocial factors [1]. These psychosocial factors can heighten vulnerability to CNP through various mechanisms, including a decreased tolerance for pain. Additionally, the anticipation or fear of pain can lead to increased pain sensitivity. There is a significant correlation between the onset and exacerbation of neuropathic disorders and psychological conditions [1]. A cross-sectional study conducted in middle-aged adults in the United Kingdom during 2017–2018 revealed that the prevalence of CNP disorders in the general population stands at 9.2%. Notably, the data indicate that the white and female demographics are more susceptible to CNP [2].

Hemifacial spasm (HS) is a type of CNP pathology. In this condition, muscles on one side of the face spasm involuntarily. The primary underlying pathology involves the breakdown of myelin at the facial nerve root entry and ephaptic transmissions [3]. Common causes of HS include vascular compression of the facial nerve, tumors, vascular malformations, and demyelinating lesions. HS affects approximately 9.8 individuals per 100,000, with the average age of onset at 44 years. There is a notably higher incidence of HS among women and Asian populations, indicating an increased sensitivity to the disorder within these groups [3].

The study by Grafe et al. indicates that women are twice as likely to experience psychological disorders, such as post-traumatic stress disorder (PTSD) and major depressive disorder (MDD), compared to men [4]. Many causes of hemifacial spasm (HS) and chronic neuropathic pain (CNP) are linked to psychosocial issues. HS intensity can escalate and be easily triggered by psychological stress. Research also shows a connection between traumatic childhood experiences and the occurrence of CNP [1]. Given the higher vulnerability of women to both psychological disorders and CNP disorders, including HS, it is plausible that underlying psychological factors may contribute to the onset and aggravation of these neuropathic conditions [2–4].

Research into psychogenic hemifacial spasms has revealed a prevalence rate of 2.4% for the condition, with all diagnosed cases occurring in women [5]. Despite the diverse causes of hemifacial spasm (HS), common pathologies include vascular malformations and tumors that exert pressure on the facial nerve [3]. Considering the scientific data, the pronounced prevalence of CNP disorders, including HS, in women may stem from a heightened vulnerability to psychological disturbances. The stress associated with HS can exacerbate symptoms, leading to increased fatigue and mood changes. This escalation can trigger further complications, creating a vicious cycle that particularly affects women, making them more susceptible to these severe neurological pain disorders. The treatment of CNP is challenging due to its complex mechanisms; therapies provide symptomatic relief but do not address the root cause [1]. In clinical practice, addressing this complexity necessitates an interdisciplinary approach that includes not only pharmacological treatments but also cognitive behavioral therapy, physical and occupational therapy [1]. This multifaceted approach underscores the necessity of incorporating psychological evaluations and support into treatment protocols.

The medical community, particularly neurologists and neurosurgeons, should prioritize screening patients with CNP disorders, including HS, for underlying psychological pathologies before proceeding to more invasive diagnostic procedures. This proactive approach includes not only early psychiatric assessments but also the implementation of de-stressing techniques and psychological support to manage their conditions effectively. Techniques such as cognitive-behavioral therapy, mindfulness practices, and stress management interventions can be vital in mitigating the psychological impacts that exacerbate CNP symptoms. Integrating these methods can help reduce the immediate reliance on costly and physically demanding procedures like nerve conduction studies, magnetic resonance imaging (MRI), and computed tomography (CT) scans [1].

Furthermore, the research community is encouraged to conduct clinical psychosocial studies to statistically explore the relationship between CNP disorders such as HS and psychological disturbances, particularly monitoring the progression in women. Such research could reveal significant gender-specific patterns and improve our understanding of the pathopsychological aspects of these disorders. Improved knowledge could lead to enhanced preventative measures for women, promoting more effective and efficient treatments and potentially sparing many from the severe sequelae of these debilitating conditions.
